# Pharmacokinetics of Molnupiravir in Cats with Naturally Occurring Feline Infectious Peritonitis

**DOI:** 10.3390/pathogens14070666

**Published:** 2025-07-07

**Authors:** Petra Černá, Luke Wittenburg, Jennifer Hawley, McKenna Willis, Britta Siegenthaler, Michael R. Lappin

**Affiliations:** 1 Department of Clinical Sciences, Colorado State University, Fort Collins, CO 80523, USA; 2 Department of Surgical and Radiological Sciences, School of Veterinary Medicine, University of California-Davis, Davis, CA 95616, USA

**Keywords:** MPV, coronavirus, antiviral therapy

## Abstract

Antiviral drugs like EIDD-2801 (molnupiravir; MPV) have been successfully used in the treatment of feline infectious peritonitis (FIP). The previous study of the pharmacokinetics of MPV in healthy cats showed promise for its use and safety. The objective was to determine the pharmacokinetics of molnupiravir in cats with naturally occurring FIP by measuring MPV and EIDD-193 (β-D-N4-hydroxycytidine; NHC) serum levels. Blood was collected from seven cats diagnosed with naturally occurring FIP treated at 1, 2, 4, 6 and 12 h post oral MPV administration and at 12 h post pill administration 7 days later. Serum concentrations of MPV and NHC were determined using a previously published high-performance liquid chromatography–tandem mass spectrometry (HPLC-MS/MS) method. The mean dose of MPV was 15.44 mg/kg (SD ± 1.82). The mean peak serum concentration of MPV (C_max_) after a single PO dose of MPV was 38 ng/mL (SD ± 5). The mean peak serum concentration of NHC (C_max_) after a single PO dose of MVP was 1551 ng/mL (SD ± 720). the time to reach NHC C_max_ (T_max_) was 2.6 h (SD ± 1.4), and the NHC elimination half-life was 1.6 h (SD ± 1.1). Minimal drug accumulation was seen in trough concentrations following twice-daily dosing for 7 days. The low MPV levels may be explained by fast conversion to its active metabolite NHC. The mean NHC concentrations at all time points were at least 4 times the reported in vitro IC_50_ for feline coronavirus strains and twice-daily dosing for seven days did not lead to drug accumulation within the serum. The results support the use of MPV in the treatment of FIP, and if therapeutic drug monitoring is to be performed, NHC should be measured.

## 1. Introduction

Feline infectious peritonitis (FIP) has been a challenge for veterinarians and a devastating disease among cats for over half a century, yet despite marked efforts and many theories, the pathogenesis of FIP is still not fully understood [1]. Feline infectious peritonitis is a clinical syndrome that is believed to arise from mutations of feline enteric coronavirus (FECV) that result in changes to viral pathogenicity, enabling FIP viruses (FIPVs) to replicate in monocytes and macrophages [1,2]. Recent studies have reported the use of drugs like the nucleoside analog GS-441524 as a successful treatment of FIP [3,4,5]. However, more treatment options for cats with FIP, especially those that relapse, are needed. Recently, a study showing PK/PD data from the antiviral drug EEID-2801 (FDA Emergency Use Authorization for the treatment of SARS-CoV-2 in humans) showed promise for its use and safety in cats [6]. This recent study of pharmacokinetic properties of molnupiravir (MPV; EIDD-2801) in healthy specific-pathogen-free cats established that orally administered MPV at 10 mg/kg achieves plasma levels greater than the established corresponding EC_50_ values. In that study, the MPV prodrug was detected in the serum of all three cats at low levels in the first 12 h post-administration, while the β-D-N4-hydroxycytidine (NHC; EIDD-1931) metabolite was detected at much higher levels between 12 and 24 h post-administration. A marked variability in the detected NHC serum concentration was present between individual cats. For one cat, the peak serum concentration for MPV and NHC occurred at the 3 h time point, with NHC detected at a 10-fold greater level than MPV. For another cat, the peak serum concentration occurred at the 1.5 h time point, with MPV nearly 100-fold below NHC. The peak serum concentration occurred for the third cat at the 9 h time point, with NHC detected to be nearly 100-fold over MPV. NHC was more consistently and quantifiably detected over the 24 h time period, aside for one cat, for which NHC was no longer detected (based on limit of detection) 12 h post-administration. MPV, however, was not consistently detected at the early time points or after the 12 h time point. This study reported no evidence of acute organ toxicity in any of the cats based on the pre- and post-treatment complete blood count (CBC) and serum biochemistry panels; however, all three of the MPV-treated cats demonstrated variable signs of nausea, including hypersalivation and/or vomiting, after the oral administration of MPV (10 mg/kg). Another study assessing at 18 cats with FIP being treated with MPV showed that molnupiravir might be an effective and safe treatment for domestic cats with FIP at a dose of 10–20 mg/kg twice daily [7]. In this study, increased serum alanine transaminase (ALT) activity was found in 3 of the 18 cats, all at days 7–9, and all cats recovered without any medications or need to stop the treatment [7]. There is also uncontrolled data from client surveys that suggests the efficacy of this product as rescue treatment following failure of the GS-441524 therapy as well as a first-line treatment option for cats with FIP [8]. A recent study evaluating MPV in cats with FIP showed that it is an effective antiviral treatment for effusive FIP, with 8/10 cats being in remission at 16 weeks, while the 2 non-survivors died in the first 24 h of treatment [9]. The survival of cats treated with oral MPV was non-inferior to that of historic control cats treated orally with GS-441524 [9].

## 2. Materials and Methods

### 2.1. Single-Dose Pharmacokinetics of Orally Administered MPV

Seven cats with naturally occurring FIP were enrolled in this in vivo PK study to determine the feline-specific pharmacokinetics and metabolism of orally administered MPV. This study was approved by the Institutional Animal Care and Use Committee (IACUC #5961). Powdered MPV (NM Pharmtech, Taian, China) was utilized for the PK study. Orally administered MPV was formulated as excipient-less powder in size 3 gelatin capsules (Monument Pharmacy, Monument, CO, USA). Physical examination was performed on each cat at study initiation (time point zero), and an IV catheter was placed into a cephalic vein, secured in place, and flushed with heparinized saline. Blood was collected from the catheter prior to MVP administration (time point 0) to establish a baseline complete blood count and serum biochemistry analysis panel for the MPV study. These panels were repeated 7 days post MPV administration to assess for toxicity. After baseline sampling, the cats were administered MVP drug, and then approximately 0.5–1 mL of whole blood was collected from the IV catheter at each of the following time points: 1, 2, 4, 6 and 12 h post administration of MPV. At each collection time point, the blood sample was incubated for 5 min at room temperature to allow for clotting and then centrifuged at 12,000× *g* for 10 min to separate the serum fraction from the blood cells. One hundred microliters of serum were aliquoted into a 1.5 mL microcentrifuge tube containing 300 μL of acetonitrile to halt compound metabolism by serum esterases and immediately placed into a −80 °C freezer prior to shipping overnight on dry ice for high-performance liquid chromatography–tandem mass spectrometry (HPLC/MS-MS) analysis. In addition, oral MPV administration was continued twice daily for seven days, and serum trough concentrations of NHC were determined on day 7, 12 h after the previous dose of MVP.

### 2.2. Liquid Chromatography–Tandem Mass Spectrometry (LCMSMS) Quantitation of Serum MPV Concentrations and Pharmacokinetic Analysis

Analysis of serum MPV and its main metabolite, NHC (EIDD-1931), was performed by modification of a previously validated and published method for human samples [10]. Briefly, stock solution of MPV (EIDD-2801; NM Pharmtech, 99.0% purity) and NHC (EIDD-1931; MedChemExpress, Monmouth Junction, NJ, USA, 99.73% purity) were prepared at 2 mg/mL in methanol and stored at −80 °C. Stock solutions were further diluted in methanol to produce calibration standards for a low concentration curve at 1, 5, 10, 50 and 100 ng/mL of MPV and NHC combined and a high concentration curve at 250, 500, 750, 1000, 2500 and 5000 ng/mL of NHC alone. Calibration curves and quality control samples were generated as described previously [6].

### 2.3. Mass Spectrometry and Liquid Chromatography Conditions

Negative ion electrospray ionization mass spectra were obtained on a Sciex 6500+ Q-TRAP triple quadrupole mass spectrometer (AB Sciex LLC, Framingham, MA, USA) with a turbo ionspray source coupled to the Sciex ExcionLC™ UHPLC system (Sciex, Marlborough, MA, USA) with a cooled (15 °C) autosampler. Chromatographic separation was carried out on a Luna^®^ Phenyl-hexyl 3.0 µm column (50 × 2.0 mm) with a filter frit guard column (both from Phenomenex, Inc., Torrance, CA, USA). A gradient mobile phase was employed consisting of 1 mM ammonium acetate pH 4.3 (mobile phase A) and acetonitrile containing 1 mM ammonium acetate (mobile phase B). Separation was carried out by holding mobile phase B constant at 2% for 1.2 min, increasing linearly to 90% at 2.2 min, holding at 90% until 3.5 min, decreasing linearly back to 2% from at 3.75 min and equilibrating at 2% until 5.0 min. Analytes were identified by monitoring the ion transitions for MPV (*m*/*z* 328.1 → 126.0 and 168.1) and for NHC (*m*/*z* 257.9 → 125.9 and 107.9). Quantitation was performed by linear regression of analyte peak areas in unknown samples with calibrator samples using 1/x^2^ weighting.

### 2.4. Pharmacokinetics Analysis

Pharmacokinetic parameters for NHC were examined following oral dosing of MPV and were estimated by noncompartmental analysis with the linear trapezoidal–linear interpolation calculation method and uniform weighting using the commercially available software program Phoenix WinNonlin v8.3 (Certara Inc., Princeton, NJ, USA). The half-life (T_1/2_) was estimated using a minimum of three time points in the elimination phase. For serum MPV, only the maximum serum concentration (C_max_), time to reach the maximum serum concentration (T_max_) and area under the serum MPV concentration–time curve from time 0 to 12 h (AUC_0-T_) were estimated. Graphs were generated using GraphPad Prism v9.1.2 (GraphPad Software, Boston, MN, USA).

## 3. Results

### 3.1. Assay Performance

The calibration curve was linear between 5 and 2000 ng/mL (r > 0.997) with a lower limit of quantitation of 5 ng/mL NHC and 2.5 ng/mL for MPV (signal to noise ratio > 10). The accuracy of calibrators was within 15% at all concentrations, and the accuracy and precision of quality control samples were also within 15% (n = 3 per concentration).

### 3.2. Safety and Tolerability of Oral Molnupiravir

The mean oral dose of MPV administered was 15.44 mg/kg (SD ± 1.82). No evidence of severe side effects or MPV-associated organ toxicity were noted in any of the cats; however, based on the pre- and post-treatment complete blood count (CBC) and serum biochemistry panels, one cat developed a mild increase in ALT activity (142 IU/L; reference interval (RI) 30–140). Moreover, one MPV-treated cat showed signs of nausea (hypersalivation) after the oral administration of MPV.

### 3.3. Pharmacokinetics of Oral MPV and NHC in Cats with FIP

**Figure 1:**
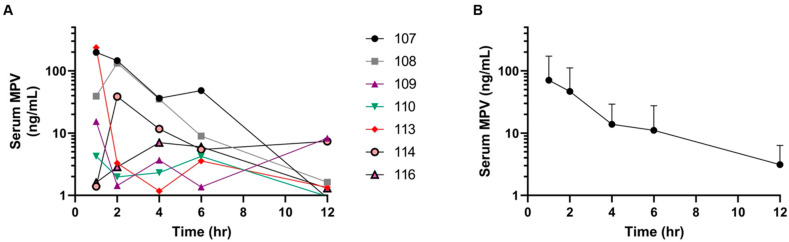
Serum concentration–time curves of the prodrug MPV over a 12 h time period in cats. (**A**) Serum MPV concentrations (ng/mL) of each individual cat over 12 h. (**B**) Combined serum MPV concentrations in all cats at 1, 2, 4, 6 and 12 h post MPV administration (black dots are arithmetic mean values; error bars represent standard deviation).

The full patient information including the age, sex, breed and FIP form can be seen in Table 1. Six out of the seven cats finished 12 weeks of therapy with MPV and are in remission now with all clinical signs resolved. One patient died 11 days post diagnosis of FIP, which was suspected to be due to FIP, but a post-mortem examination was not performed. The MPV prodrug was detected in the serum of all seven cats at low concentrations, with 5 of 7 cats having serum concentrations above the limit of quantitation at 12 h post-administration (Figure 1A). There was notable interpatient variability in MPV concentrations at each time point, as demonstrated in Figure 1B. Select PK parameters for MPV are depicted in Table 2. Following the oral administration of MPV, serum C_max_ was extremely variable, with a range of 4 to 236 ng/mL (geometric mean of 37 ± 5 ng/mL). The time to reach the maximum serum concentration ranged from 1 h (4 of 7 cats) to 4 h (1 cat) with a mean of 1.7 h. Total MPV exposure from time zero to 12 h (AUC_0-T_) ranged from 32 to 685 h·ng/mL with a geometric mean of 131 ± 3 ng/mL.

**Figure 2:**
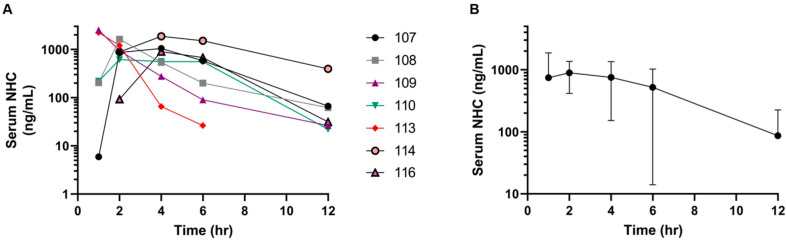
Serum concentration–time curves of the active metabolite NHC over a 12 h time period in cats. (**A**) Serum NHC concentrations (ng/mL) of each individual cat over 12 h. (**B**) Combined serum NHC concentrations in all cats at 1, 2, 4, 6 and 12 h post MPV administration (black dots are arithmetic mean values; error bars represent standard deviation).

The arithmetic mean serum concentration–time profiles of the active metabolite, NHC, are presented in Figure 2. NHC was detectable at the first measured time point (1 h) and was detectable out to 12 h in all cats. Estimated PK parameters for NHC are presented in Table 2. C_max_ for NHC ranged from 617 to 2530 ng/mL (mean of 1551 ± 720 ng/mL), which is approximately 6 µM. The time to reach the maximum serum NHC concentration was longer than that of MPV, with a mean ± SD T_max_ of 2.6 ± 1.4 h. Total exposure to NCH was also quite variable and ranged from 4222 to 12,323 h·ng/mL (mean ± SD 5919 ± 2873 h·ng/mL). The harmonic mean serum half-life (T_1/2_) for NHC was 1.6 ± 1.1 h. Average NHC concentrations at all time points were at least 4 times the reported in vitro EC_50_ for feline coronavirus strains: 0.05 μM for Black I (serotype I FIPV) and 0.11 μM for WSU-79-1146 (serotype II FIPV) [6].

**Figure 3:**
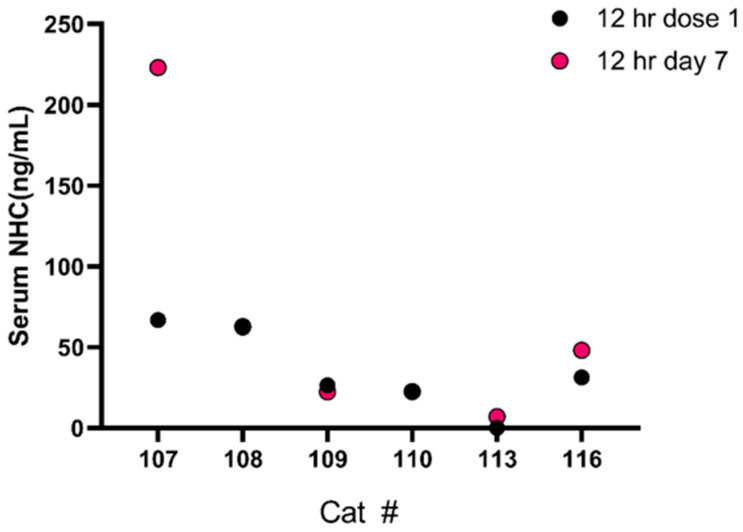
Comparison of serum detection of the metabolite NHC at 12 h time point in six cats after one dose and at day 7. Note that cats 108 and 110 have identical 12 h sampling at days 1 and 7.

Six of the cats had serum NHC concentrations measured again on day 7 at 12 h post-dose. In 5/6 cats the trough serum NHC concentrations were very similar between days 1 and 7; however, in one cat the day 7 concentration was much higher than it was following the day 1 dose (66.8 ng/mL on day 1 vs. 223 ng/mL) (Figure 3). The accumulation ratio for trough serum NHC concentrations ranged from 0.8 to 3.3 (mean 1.5 ± 0.9).

## 4. Discussion

Molnupiravir is an oral prodrug of the broadly active, antiviral ribonucleoside analog *N4*-hydroxycytidine (NHC). The primary circulating metabolite NHC is taken up into cells and phosphorylated to NHC-triphosphate (NHC-TP) [11]. NHC-TP serves as a competitive substrate for viral RNA-dependent RNA polymerase (RdRp), which results in an accumulation of errors in the viral genome, rendering virus replication ineffective. Molnupiravir has demonstrated activity against SARS-CoV-2 and FCoV. Little to no molnupiravir is observed in plasma due to rapid hydrolysis to NHC, which was found in this study as well. MPV is rapidly metabolized in cat serum to the active metabolite, NHC [12]. In our study, the NHC metabolite was detected at early time points and at markedly higher concentrations than the administered prodrug, MPV.

The mean NHC concentrations at all time points were at least 4 times the reported in vitro IC_50_ for feline coronavirus strains, and this study also found interindividual variability in MPV metabolism (Figure 1 and Figure 2). This is similar to the previous study in healthy cats, and a definitive cause for this metabolic variability is not clear; however, it is possible that the metabolism could have been affected by the systemic FIP disease [6]. We only performed PK in seven cats, and this small group might have been a limitation to the study; however, a previously published study in three healthy cats showed similar interindividual variability [6]. It has been suggested that variation in genetic polymorphisms related to metabolic pathways, such as the cytochrome P450 system, might also play a role in this variability [13]. Moreover, a minor variability in the timing between centrifugation of individual whole blood samples between cats and the timing of the transfer of feline serum into acetonitrile could also contribute to the variability in measured concentrations of MPV and/or NHC.

The mean maximum serum concentration in this study was 37 ± 5, which is higher than previously reported in the healthy cats administered MPV 14.2 ± 13.8, which is likely due to the fact that these cats had systemic FIP disease. An estimated NHC half-life of approximately 3 h was previously reported, while our current study estimated an elimination half-life of 1.6 h [6]. Moreover, twice-daily dosing for seven days did not lead to substantial drug accumulation within the serum in the majority of the cats. The one cat that had higher NHC levels at day 7 was a cat with severely increased total bilirubin levels (1.5 mg/dL; RI (0–0.1)) which could have led to reduced excretion and thus accumulation of the drug in serum. A similar lack of NHC accumulation following twice-daily dosing of MPV in humans has been reported; however, this same study did detect a greater degree of accumulation of intracellular NHC-TP in PBMCs, which was not measured in this study [14]. No evidence of acute toxicity in the MPV-treated cats based on complete blood count and blood chemistry was found apart from one cat having a mild increase in ALT activity and one cat having mild nausea post-administration. The associations between the administration of MPV and nausea in cats and increased liver enzyme activities has been reported in other studies [6,7]. Moreover, the cats were also fasted prior to receiving MPV, which might have played a role in the development of nausea. In humans, there was a food effect on the rate of absorption observed after MPV administration; however, the therapeutic exposures during fasted and fed states were comparable [12].

There are several published clinical trials focused on the treatment of cats with naturally occurring FIP [9,15]; however, most focused on the treatment of cats without therapeutic drug monitoring or pharmacokinetic studies. There are also no studies looking at the penetration of antiviral compounds into feline tissues, and this study did not evaluate this, which is a limitation of this study. Additional in vivo pharmacokinetic studies, assessment of compound penetration into tissues, and rigorously designed, prospective, focused clinical trials are needed for all drugs used, including MPV. In addition, the optimal combination antiviral therapies and the compound’s ability to penetrate cerebrospinal fluid and aqueous humor need to be considered in order to optimize therapeutic approaches in cats with ocular or neurological FIP and particularly those that experience therapeutic relapse. Measurements of drug levels in serum but also assessments of absorption in tissues, cerebrospinal fluid and aqueous humor are important to establishing the best dosing protocols of molnupiravir in cats, further optimizing and individualizing the treatment cats with FIP, and providing better therapeutic drug monitoring recommendations for these cats. Further analysis of therapeutic drug monitoring for response to treatment is needed because a recent study showed that assessment of the predictive relationship between the median GS-441524 plasma trough concentration and achieving simple remission failed to demonstrate a significant correlation [16].

## 5. Conclusions

In conclusion, we have established pharmacokinetic analysis of molnupiravir in cats with naturally occurring FIP, which showed early metabolization to NHC and interindividual variability in MPV metabolism. The mean NHC concentrations at all time points were at least 4 times the reported in vitro IC50 for feline coronavirus strains, and twice-daily dosing for seven days did not lead to drug accumulation within the serum.

## Figures and Tables

**Table 1:** Patient information on age, sex, breed, FIP form, and the outcome for individual cats.

Number	Age (Years)	Sex	Breed	FIP Form	Outcome
107	0.34	MN	DSH	effusive	dead (day 10)
108	3.01	MN	DSH	effusive	in remission
109	1.06	MN	DSH	effusive	in remission
110	1.45	FS	Maine Coon	ocular	in remission
113	0.43	MN	DSH	non-effusive	in remission
114	1.08	MN	DSH	effusive	in remission
116	2.00	FS	Bengal	effusive	in remission

MN—male neutered, FS—female spayed, DSH—domestic shorthair.

**Table 2:** Pharmacokinetic estimates for MPV and NHC following a single oral dose of molnupiravir in seven cats with FIP.

	MPV		NHC	
Parameter	Mean ± SD	Median (Range)	Mean ± SD	Median (Range)
C_max_ (ng/mL)	37 ± 5 ^†^	39 (232)	1551 ± 720	1630 (1913)
T_max_ (h)	1.7 ± 1.1	1 (3)	2.6 ± 1.4	2 (3)
AUC_0-T_ (h·ng/mL)	222 ± 237	127 (653)	5919 ± 2874	4749 (8100)
AUC_0-Inf_ (h·ng/mL)			6296 ± 3573	4957 (10,040)
K_el_ (1/h)			0.344 ± 0.152 ^‡^	0.35 (0.76)
T_1/2_ (h)			1.6 ± 1.1 ^‡^	1.9 (2.7)
Vz/F (L)			24.4 ± 11.3	23.5 (30.6)
Cl/F (L/h)			9.2 ± 3.9	10.4 (9.8)

C_max_: maximum serum concentration; AUC_0-T_: area under the serum concentration–time curve from time 0 to 12 h; AUC_0-Inf_: area under the serum concentration–time curve from time 0 extrapolated to infinity; K_el_: elimination rate constant; T_1/2_: serum half-life; Vz/F: apparent volume of distribution; CI/F: apparent clearance. ^†^ Geometric mean with geometric standard deviation; ^‡^ harmonic mean with pseudo-standard deviation.

## Data Availability

The original contributions presented in this study are included in the article. Further inquiries can be directed to the corresponding author.

## Abbreviations

The following abbreviations are used in this manuscript:

MPV	molnupiravir
NHC	*N4*-hydroxycytidine
C_max_	peak serum concentration
